# Heterologous Overexpression of NtNACa from *Narcissus tazetta* L. var. *chinensis* ‘Yunxiang’ Enhances Drought and Salt Stress Resistance in *Arabidopsis thaliana*

**DOI:** 10.3390/genes17030316

**Published:** 2026-03-13

**Authors:** Peng-Fei Li, Yong Wu, Xiang-Yun Rui, Xiao-Jing Chen, Ming-Yue Wei, Huan Li

**Affiliations:** 1 College of Biotechnology and Bioengineering, Zhejiang University of Technology, Hangzhou 310014, China; lipengfei@stu.xmu.edu.cn; 2 Moganshan Institute ZJUT, Huzhou 313200, China; 3 Xiamen Ruidu Biotechnology Co., Ltd., Xiamen 361100, China; yorkwu724@126.com; 4 College of Food and Bio-Engineering, Bengbu University, Bengbu 233030, China; rxy@bbc.edu.cn; 5 College of Horticulture, Fujian Agriculture and Forestry University, Fuzhou 350002, China; xjchen804@sina.com; 6 School of Ecology, Resources and Environment, Dezhou University, Dezhou 253000, China

**Keywords:** abiotic stress tolerance, gene cloning, expression analysis, functional characterization, subcellular localization, drought tolerance, NACa

## Abstract

Background/Objectives: NAC transcription factors are key regulators of stress responses, yet their roles in *Narcissus tazetta* L. var. *chinensis* remain uncharacterized. This study aimed to isolate and functionally analyze *NtNACa*, a *NAC* gene from the ‘Yunxiang’ narcissus variety, to evaluate its potential in enhancing abiotic stress tolerance. Methods: *NtNACa* was cloned and its expression pattern under heat, salt, and ABA treatments was assessed via qRT-PCR. Subcellular localization was determined using GFP fusion in tobacco. *NtNACa* was overexpressed in *Arabidopsis thaliana* through floral dip transformation, and transgenic lines were subjected to NaCl, ABA, and drought stress assays. Results: The results showed that *NtNACa* has high homology with monocot NAC family members and possesses typical NAC transcription factor features. Further analyses revealed that *NtNACa* localizes to the nucleus, and tissue-specific expression analysis indicated that it is highly expressed in leaves, followed by roots and bulbs. The transcriptional expression of *NtNACa* is differentially regulated in response to 100 mM NaCl, 100 μM ABA, and 50 °C temperature stress. Overexpression of *NtNACa* in *A. thaliana* produced transgenic lines with significantly higher germination rates under ABA and NaCl treatments. Soil-grown transgenic *A. thaliana* plants overexpressing *NtNACa* showed markedly increased drought stress. Moreover, *NtNACa* confers drought resilience by coordinately suppressing oxidative damage (via reduced O_2_^−^· production rate and MDA accumulation and elevated *AtCAT2* expression), enhancing osmotic adjustment (through *AtP5CR*-mediated proline biosynthesis), and activating core stress-signaling components such as *AtRD29A* and *AtSnRK2.4*. Conclusions: Taken together, these results indicate that heterologous overexpression of *NtNACa* from ‘Yunxiang’ (*N. tazetta*) confers enhanced drought and salt tolerance in *A. thaliana*.

## 1. Introduction

Among the environmental factors affecting plant growth, water availability is paramount [1]. Drought impacts plant growth via physiological and biochemical processes-including photosynthesis, respiration, mineral absorption, nutrient metabolism, and hormone regulation—ultimately reducing biomass or yield [2,3,4]. As the most common abiotic stress, drought causes greater yield losses than all pests and diseases [5], with global annual losses averaging $23 billion since the 1970s [6]. Plants have evolved diverse drought-responding mechanisms. Under stress conditions, rhizobacteria promote the accumulation of abscisic acid and osmotic regulators, thereby enhancing plant stress tolerance [7]. Mild drought triggers stomatal regulation, adjusts assimilate distribution, and improves water uptake to balance transpiration and growth [8]. Severe drought boosts antioxidant enzyme activity to scavenge reactive oxygen species and maintain homeostasis [9]. Plants also synthesize osmoprotectants (e.g., mannitol, sorbitol, soluble sugars, proline) to reduce cellular water loss and preserve function [10,11]. While drought is commonly perceived as reducing biomass and yield through impairments in photosynthesis, nutrient uptake, and hormonal imbalance, recent advances reveal a more nuanced perspective. Moderate water limitation, when precisely managed, can act as a deliberate elicitor to modulate plant metabolism and enhance desirable quality traits, such as the accumulation of antioxidants and osmoprotectants, without significantly affecting growth or productivity [12]. In this context, the selection of drought severity becomes critical. Moreover, the efficacy of such approaches is highly species- and cultivar-dependent, necessitating the identification of key regulatory genes.

Transcription factors (TFs), or trans-acting factors, are proteins that specifically bind to cis-acting elements in eukaryotic gene promoters, regulating downstream target gene expression spatially and temporally to enable plants to adapt to complex environments. These proteins have four main functional regions (DNA-binding domain, transcription regulation domain, nuclear localization signal (NLS) and oligomerization site) and are categorized by DNA-binding domains into families like NAC, MYB, WRKY, bZIP, and AP2/ERF. NAC TFs feature a conserved N-terminal protein binding domain (PBD, with subdomains A–E involved in nuclear localization and target DNA recognition) and a variable C-terminal transcription regulatory region (TRR, with activation or repression roles) [13]. These factors bind downstream gene promoters, triggering cascades via gene regulation, with three regulatory levels: transcriptional (via stress-responsive cis-elements like LTREs, ABREs, and W-Box in NAC promoters) [14,15], post-transcriptional (miRNA-mediated target degradation) [15,16], and post-translational (dimerization, ubiquitin-mediated degradation, and interactions with other proteins) [15]. Studies have shown that NAC TFs are key in plant stress response networks and hold promise for enhancing stress tolerance. Overexpressing soybean *GmNAC11/20* in *Arabidopsis* improved salt and freeze tolerance and promoted lateral root growth [17]. Compared to wild-type, *OsNAC10*-transgenic rice demonstrated greater drought tolerance with yield increases of 25–42% under drought stress, while maintaining 5–14% higher yield under normal conditions [18]. The *NAC* gene *SHAT1-5* activated secondary wall biosynthesis, with expression levels 15-fold higher than the wild-type allele, by disrupting upstream repressors, thereby improving legume traits [19]. Studies have also been conducted on NAC transcription factors in plants such as *Glycyrrhiza uralensis* [20], *Morus atropurpurea* [21], and *Acer palmatum* [22], making NACs a research hotspot in plant stress resistance. However, in-depth domestic studies focus on model plants, major cash crops, and a few vegetables and flowers, with limited reports on *NAC* transgenic studies involving *N. tazetta* and other bulbous flowers.

The ‘Yunxiang’ Narcissus, a variety of *N. tazetta* bred by the Institute of Horticultural Genetics and Breeding, Fujian Agriculture and Forestry University, has expanded the range of *N. tazetta* varieties to some extent. It exhibits excellent disease resistance and stress tolerance, blooming in cold winters. Since NAC transcription factors play key roles in plant stress response and development, this study aimed to clone a NAC transcription factor gene, *NtNACa*, from the ‘Yunxiang’ narcissus and to functionally characterize its role in abiotic stress responses through heterologous expression in *Arabidopsis thaliana*. The findings are expected to provide theoretical insights and a candidate gene for future molecular breeding of stress-tolerant narcissus cultivars.

## 2. Materials and Methods

### 2.1. Narcissus Cultivation Conditions

Narcissus bulbs were provided by the Institute of Horticultural Genetic Breeding, Fujian Agriculture and Forestry University (Fuzhou, China). This study was conducted in the laboratory of omics technology and bioinformatics at Bengbu University. Uniform three-year-old narcissus plants with a diameter of approximately 6.5 cm were selected and surface-sterilized with 75% ethanol and 10% sodium hypochlorite (10 min), followed by 4–5 rinses with ddH_2_O. The plants were subsequently cultured in Hoagland nutrient solution under controlled environmental conditions: temperature of 28 °C, relative humidity of 75%, and a photosynthetically active radiation intensity of 800–1000 μmol m^−2^ s^−1^. When the leaves grew to a length of 8 cm, plants with uniform leaf lengths were used for the experiments. Each treatment included a total of 20 narcissus plants, arranged in five biological replicates, each replicate with four seedlings. The bulbs were divided into four treatment groups: three groups cultured in Hoagland solution (control), 100 μM ABA, or 100 mM NaCl at 28 °C (16 h/8 h light/dark), and one group was subjected to a heat shock treatment by incubation in Hoagland solution at 50 °C (same photoperiod). Similar extreme temperature was selected to elicit a strong and rapid transcriptional response of stress-related genes [23,24]. Bulbs were placed in open containers to allow gas exchange, minimizing potential hypoxia during the short-term exposure (sampling up to 24 h). Leaf samples were collected at 0, 1, 3, 6, 12, and 24 h post-treatment, flash-frozen in liquid nitrogen, and stored at −80 °C for analysis. For each time point under every stress treatment, leaf samples were collected from independent sets of plants (destructive sampling), with each biological replicate consisting of pooled leaves from four individual seedlings.

### 2.2. Gene Cloning and qRT-PCR Analysis

Total RNA was isolated from samples using the FastPure Universal Plant Total RNA Isolation Kit (Vazyme, Nanjing, China) according to the manufacturer’s protocol. Within each biological replicate, leaf tissues from multiple seedlings were pooled before RNA extraction to obtain adequate material, with each replicate processed independently. RNA quality was assessed by measuring concentration with a microspectrophotometer and verifying integrity through 1% agarose gel electrophoresis. Subsequently, 500 ng of total RNA was reverse-transcribed into cDNA using the PrimeScript^TM^ RT Reagent Kit with gDNA Eraser (Takara, Kusatsu, Japan). The synthesized cDNA was then diluted 1:2 with nuclease-free ddH_2_O and homogenized by vortex mixing.

PCR amplification of the *NtNACa* gene was carried out using synthesized cDNA as template and gene-specific primers *NtNACa*-F (5′-GGACAACAAATTCTCGATAAGCTA-3′) and *NtNACa*-R (5′-CGCCTGTAATCAATGATGTTTC-3′), with ApexHF HS DNA Polymerase (Accurate Biology, Changsha, China) according to the manufacturer’s recommended cycling conditions.

For quantitative analysis, qRT-PCR was conducted using SYBR Premix Ex Taq^TM^ (Perfect Real Time, Takara, Kusatsu, Japan) on a real-time PCR system. Gene expression levels of NACa were determined using the primer pair *NtNACa*-qPCR-F (5′-AGCAGGCATCTCGGAATCAAG-3′) and *NtNACa*-qPCR-R (5′-CGGCACAGAACCCAGTCATC-3′). *NtActin* (GenBank: JN204912.1) from *N. tazetta* var. *chinensis* with primers *NtActin*-qPCR-F (TGCCCAGAAGTGCTATTCCAG) and *NtActin*-qPCR-R (GTTGACCCACCACTAAGAACAATG) was used as the internal reference gene [25]. The qRT-PCR analysis was performed in a 25 μL system comprising 12.5 μL of 2× Premix ExTaq™ (Takara, Kusatsu, Japan), 9.5 μL of ddH_2_O, 0.5 μL each of the forward and reverse primers (*NtNACa*-qPCR-F/R), and 2 μL of cDNA. The thermal cycling procedure entailed a 3-min pre-denaturation at 94 °C, succeeded by 40 cycles of denaturation (94 °C, 15 s), annealing (60 °C, 15 s), and extension (72 °C, 20 s). Ct values were acquired from the melting and amplification curves, and the relative expression of genes was quantified using the 2^−ΔΔCT^ method [26].

### 2.3. Construction of pCAMBIA1300::NtNACa::GFP Recombinant Vector and Subcellular Localization Analysis in Tobacco

The pCAMBIA1300-GFP vector was digested with SacI/SalI (QuickCut^TM^, Takara, Japan) and verified by electrophoresis. Homologous arms were added to *NtNACa* via PCR (PrimeSTAR^®^ Max, Takara, Kusatsu, Japan) using designed primers (*NtNACa*-SacI-F: 5′-gagaacacgggggacgagctcATGATGATGATGACGGTACCTACG-3′, *NtNACa*-SalI-R: 5′-gcccttgctcaccatgtcgacGAAATGGGGGAGGATACTAGTCTG-3′) within CE Design software (v1.0.7, Vazyme, Nanjing, China). The digested vector and *NtNACa* fragment were gel-purified (FastPure Kit, Vazyme, Nanjing, China) and ligated (ClonExpress II, Vazyme, Nanjing, China) to generate pCAMBIA1300::*NtNACa*::GFP, which was transformed into *Escherichia coli* and sequenced.

For subcellular localization analysis, the recombinant vector was introduced into *Agrobacterium tumefaciens* strain GV3101 via heat shock transformation [27]. The transformed strain and Agrobacterium carrying the nuclear marker gene (35S::H3::mCherry) were separately cultured in Luria–Bertani (LB) medium to an OD600 = 0.6. Bacterial cells were pelleted by centrifugation and resuspended in infiltration buffer (10 mM MES, 10 μM MgCl_2_, 200 μM acetosyringone, pH 5.6), followed by incubation at room temperature for 2–3 h. Approximately one-month-old tobacco plants with fully expanded leaves were agroinfiltrated and maintained at 25 °C (12 h light/dark) for 48 h [28]. Temporary leaf sections were prepared and examined under a confocal laser scanning microscope (Olympus FV3000, Tokyo, Japan) for image acquisition.

### 2.4. Floral Dip Transformation of A. thaliana with NtNACa-GFP Construct and Selection of Homozygous Transgenic Lines

The *A. thaliana* transformation was performed using a modified floral dip protocol [29]. The pCAMBIA1300::*NtNACa*::GFP vector was introduced into *A. tumefaciens* GV3101 by heat shock. Bacteria were cultured in LB medium to OD600 = 0.6, pelleted, and resuspended in infiltration medium (4.33 g/L MS salts, 5% sucrose, 10 μL Silwet-77, pH 5.6). After 2–3 h incubation, 4–5-week-old *Arabidopsis* inflorescences (with mature siliques removed) were dipped in the suspension for 5 min, then kept in darkness (22 °C, 24 h) before transfer to normal growth conditions (25 °C, 12 h light). Mature seeds collected from these plants constituted the T0 generation transformants.

T0 generation seeds were surface-sterilized using 75% ethanol for 5 min, followed by cold stratification at 4 °C for 48 h. The treated seeds were then plated on selection medium containing 20 mg/L hygromycin. After 7 to 10 days of cultivation, resistant seedlings were transferred to soil. Genomic DNA was extracted using the TaKaRa MiniBEST kit (Takara, Kusatsu, Japan) and analyzed by PCR with *NAC*-specific primers to confirm T1 transformants. Homozygous T3 lines were established based on complete hygromycin resistance observed in all progeny.

### 2.5. ABA and NaCl Stress Assays in Wild-Type and Transgenic A. thaliana

For ABA and NaCl treatments, half-strength Murashige and Skoog (½ MS) medium was supplemented with 2 μM ABA or 100 mM NaCl. Each treatment includes three biological replicates. Each plate was divided into four equal sectors, with each sector containing 16 *Arabidopsis* seeds. Wild-type and transgenic *Arabidopsis* seeds were surface-sterilized by treatment with 75% (*v*/*v*) ethanol for 5 min, followed by five washes with ddH_2_O. The seeds were then evenly distributed in designated sectors and cultured under greenhouse conditions (22 ± 1 °C, 60% relative humidity, 16/8 h light/dark cycle). After one week of cultivation, the growth phenotypes of wild-type and transgenic *Arabidopsis* under treatment with 2 μM ABA or 100 mM NaCl were observed, and the expression level of *NtNACa* in transgenic plants under the same treatments was analyzed by qRT-PCR. Germination percentage was calculated per plate (i.e., per biological replicate) as the number of germinated seeds divided by the total number of seeds (*n* = 16) on that plate.

### 2.6. Drought Stress Assays in Wild-Type and Transgenic A. thaliana

For drought stress treatment, the culture substrate was prepared by mixing nutrient soil and vermiculite in a 1:1 ratio (*v*/*v*). An appropriate amount of water was added until the mixture reached a consistency where it could form a ball when squeezed by hand but would crumble easily upon release. The homogenized substrate was then uniformly dispensed into 12-well seedling trays (each well measuring 3.8 cm in length × 3.8 cm in width × 5 cm in height). *A. thaliana* seedlings, grown to 20 days after sowing in petri dishes, were transplanted into the trays, with one seedling per well. Subsequently, the tray cover was placed, and the seedlings were cultivated in a greenhouse (under a 16/8 h light/dark photoperiod, with a light intensity of 9000 lx and 55% relative humidity) for recovery. After five days, the cover was removed, and watering was ceased to initiate drought treatment. During this period, the growth status of the plants was continuously observed and recorded. Based on preliminary soil moisture monitoring, the gravimetric soil water content declined to approximately 7% after 17 days of water withdrawal, corresponding to 20% of field capacity. Each treatment consisted of three *Arabidopsis* seedlings, with five independent biological replicates. Survival percentage was calculated per biological replicate, defined as the proportion of surviving seedlings among the three plants within each replicate.

The rate of superoxide radical (O_2_^−^·) generation was assessed using a colorimetric assay. Briefly, 0.1 g of drought-stressed wild-type (WT) and NtNACa-overexpressing *A. thaliana* lines (OE#1, OE#2, OE#6) was homogenized in 1 mL ice-cold 50 mM phosphate buffer (pH 7.8), centrifuged at 12,000× *g* for 20 min at 4 °C, and 0.5 mL supernatant was mixed with 0.5 mL phosphate buffer and 1.5 mL of 1 mM hydroxylamine hydrochloride. After 1 h incubation at 25 °C, 2 mL of 17 mM p-aminobenzenesulfonic acid and 2 mL of 7 mM α-naphthylamine were added, and the reaction proceeded for 20 min at 25 °C. Absorbance was read at 530 nm, and O_2_^−^· production was quantified against a standard curve prepared with NaNO_2_ [30].

Malondialdehyde (MDA) content was measured using the thiobarbituric acid (TBA) assay [31]. Then, 0.1 g of WT and *NtNACa*-overexpressing *A. thaliana* lines (OE#1, OE#2, and OE#6) under drought stress was ground in 5 mL of 10% trichloroacetic acid (TCA) on ice, centrifuged at 10,000× *g* for 20 min, and 2 mL supernatant was mixed with 2 mL of 10% TCA containing 0.6% TBA. The mixture was heated at 95 °C for 15 min, cooled on ice, and re-centrifuged (10,000 rpm, 4 °C, 5 min). MDA concentration was calculated from absorbance at 450, 532, and 600 nm.

Proline content was determined using the Proline Assay Kit from Nanjing Jiancheng Bioengineering Institute (Nanjing, China), following the manufacturer’s instructions.

qRT-PCR was performed on WT and *NtNACa*-overexpressing *A. thaliana* lines (OE#1, OE#2, and OE#6) subjected to drought stress, following the protocol described in Section 2.2. Gene-specific primers were used for the following target genes: *AtRD29A* (F: ACTCAAGTGGCGGGAACTGT, R: GTAACTTCGTCGTCACGGCAG), *AtSLAC1* (F: GCTTACCGGGAGGAAACAACT, R: CAACATCTTCGCTACGGCATC), *AtAREB1* (F: GTGGTGGTCTTGTGGGACTTG, R: CCTTCTGATGACAATGGCGTAA), *AtP5CR* (F: TTCATAAGGGTGATGCCTAATACA, R: CAGCTACTCCTCCATCGGCTA), *AtSnRK2.4* (F: CTCTTGGATGGAAGTCCTGCTC, R: TCCTGGTCTTCAAATGGGTATG), and *AtCAT2* (F: ATGCACAGGGACGAGGAGGT, R: GAACAGACAGCAGGCGGAGT). *AtActin2* was used as the internal reference gene (F: CAGATGCCCAGAAGTCTTGTTC, R: TTGCTCATACGGTCAGCGATA) [32].

### 2.7. Statistical Analysis

In this study, gene expression levels and survival rates of *A. thaliana* were statistically analyzed by one-way ANOVA using SPSS software (Version 21.0), with significance level of *p* < 0.05. Error bars indicate mean values ± SD. Graphical representation and data visualization were generated using GraphPad Prism (Version 9.0.0).

## 3. Results

### 3.1. Tissue-Specific Expression of the NtNACa Gene in ‘Yunxiang’ Narcissus

**Figure 1:**
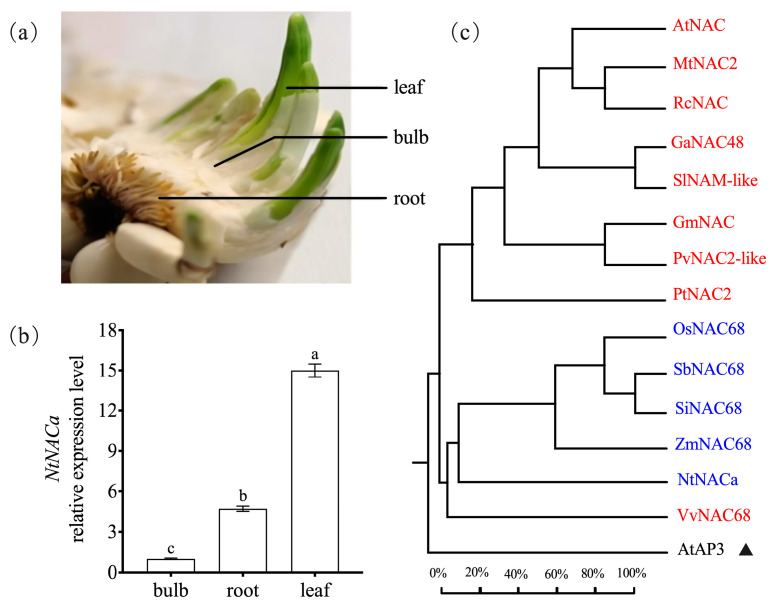
Expression pattern of *NtNACa* in different tissues of ‘Yunxiang’ narcissus and phylogram of NAC proteins from various species. (**a**,**b**) Expression pattern of *NtNACa* in bulb, root and leaf of ‘Yunxiang’ Narcissus. Data are presented as mean values ± SD (*n* = 5). Different letters on vertical bars indicate significant difference at *p* < 0.05 using one-way ANOVA. (**c**) Phylogram of NAC proteins from different species and one orthologous protein in *Arabidopsis thaliana*. AtNAC (*A. thaliana*, NP_171677.1), CaNAC48 (*Capsicum annuum*, PHT78815.1), SlNAM-like (*Solanum lycopersicum*, NP_001234219.1), PtNAC2 (*Populus trichocarpa*, XP_002306280.1), VvNAC68 (*Vitis vinifera*, XP_002283807.1), GmNAC (*Glycine max*, AGO14646.1), MtNAC2 (*Medicago truncatula*, XP_003602038.1), PvNAC2-like (*Phaseolus vulgaris*, XP_068471936.1), RcNAC (*Ricinus communis*, EEF42127.1), SbNAC68 (*Sorghum bicolor*, XP_002458677.1), ZmNAC68 (*Zea mays*, PWZ30143.1), SiNAC68 (*Setaria italica*, XP_004970362.1), OsNAC68 (*Oryza sativa*, NP_001396042.1), AtAP3 (*A. thaliana*, OAP05456.1), NtNACa (*Narcissus tazetta*, NW12212.1). In Figure 1c, black text represents the orthologous protein in *A. thaliana* used to root the evolutionary tree, blue text represents monocot NAC proteins, and red text represents dicot NAC proteins.

To investigate the potential role of the *NtNACa* gene in ‘Yunxiang’ Narcissus under stress conditions, we first examined its tissue-specific expression profiles in leaves, bulbs, and roots (Figure 1a). qRT-PCR analysis revealed significant tissue-specific variation in *NtNACa* expression, with the highest transcript abundance detected in leaves, moderate levels in roots, and the lowest expression in bulbs (Figure 1b).

### 3.2. Phylogenetic Analysis and Sequence Homology of NtNACa Proteins

Phylogenetic analysis demonstrated that NtNACa forms a well-defined clade with stress-responsive NAC proteins from monocot species, including ZmNAC68 (*Zea mays*), OsNAC68 (*Oryza sativa*), SbNAC68 (*Sorghum bicolor*), and SiNAC68 (*Setaria italica*) (Figure 1c).

**Figure 2:**
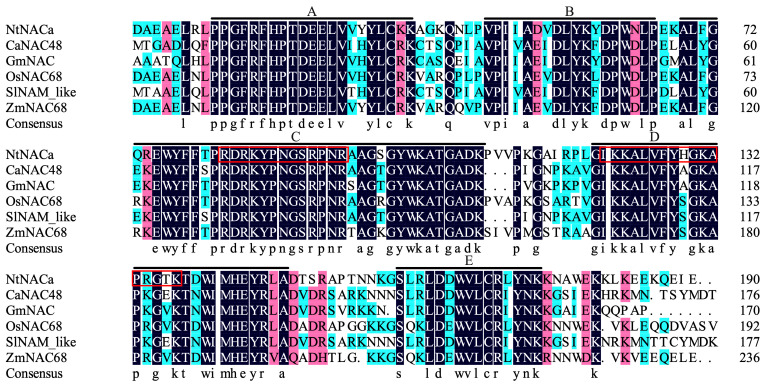
Amino acid sequence alignment of NtNACa. Multiple sequence alignment of NtNACa with NAC proteins in other species. The amino acid residues in black indicate 100% similarity, those in pink indicate 75–99% identity, and those in light blue indicate 50–74% identity. The five NAC motifs (**A**–**E**) are market. Two predicted nuclear localization signal (NLS) sequences are in red frames.

Protein sequence alignment revealed NtNACa possesses the five characteristic NAC family motifs (A–E). Importantly, we identified two putative NLSs (Figure 2, red boxes) within the C-terminal regulatory domain (Motifs C and D). These structural features support NtNACa’s nuclear targeting capability and its potential role in stress-responsive transcriptional regulation.

### 3.3. Subcellular Localization of NtNACa

**Figure 3:**
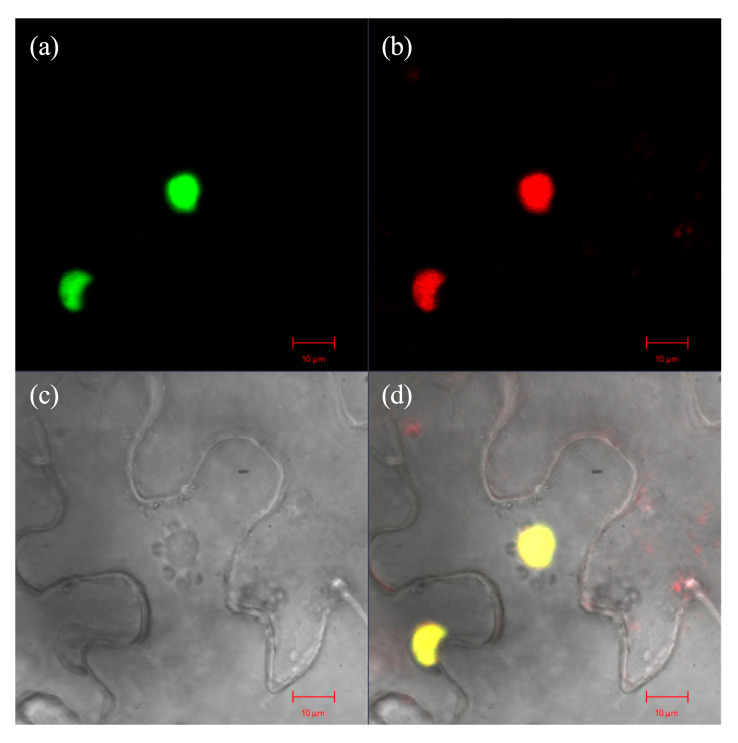
Subcellular localization of NtNACa in *Nicotiana benthamiana* epidermal cells. Confocal microscopy images show (**a**) GFP fluorescence signal with green color of NtNACa-eGFP, (**b**) mCherry fluorescence signal with red color from nuclear-localized histone H3 (H3-mCherry), (**c**) bright-field image, and (**d**) merged image with yellow color demonstrating nuclear localization of NtNACa. Scale bar: 10 μm.

Confocal microscopy analysis of *Nicotiana benthamiana* leaf cells transiently expressing the NtNACa-eGFP fusion protein demonstrated clear nuclear localization. The green fluorescence signal of NtNACa-eGFP (Figure 3a) exhibited perfect colocalization with the red fluorescence signal from the nuclear marker H3-mCherry (Figure 3b), as evidenced by the merged image (Figure 3d). Bright-field microscopy (Figure 3c) confirmed normal cellular morphology throughout the observation. Quantitative fluorescence colocalization analysis verified that NtNACa-eGFP was exclusively localized to the nucleus.

### 3.4. Transcriptional Response of NtNACa to Heat, NaCl, and ABA Treatments

**Figure 4:**
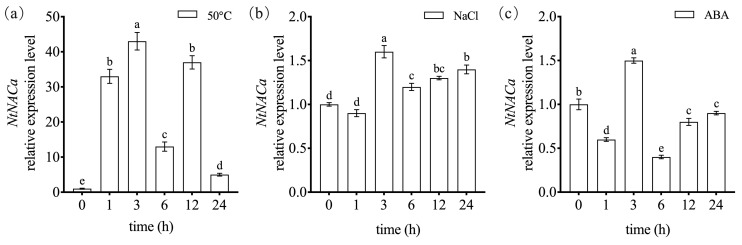
The relative expression level of *NtNACa* in leaves of ‘Yunxiang’ narcissus under 50 °C (**a**), 100 mM NaCl (**b**) and 100 μM ABA (**c**). Data are presented as mean values ± SD (*n* = 5). Different letters on vertical bars indicate significant difference at *p* < 0.05 using one-way ANOVA.

To characterize the stress-responsive properties of the *NtNACa* gene, we performed time-course expression analyses under heat, salt, and ABA treatments. Exposure to 50 °C heat stress resulted in sustained upregulation of *NtNACa* transcripts throughout the 24 h experimental period, with maximum induction occurring at 3 h (Figure 4a). NaCl treatment similarly induced *NtNACa* expression, showing significant elevation from 3 to 24 h with peak transcript accumulation at 3 h (Figure 4b). ABA treatment produced a distinct temporal response pattern: transient upregulation at 3 h was followed by significant downregulation at all subsequent time points (1, 6, 12, and 24 h) relative to the untreated control (Figure 4c).

### 3.5. Stress Response Phenotypes and Transcriptional Regulation in NtNACa-Overexpressing Arabidopsis

**Figure 5:**
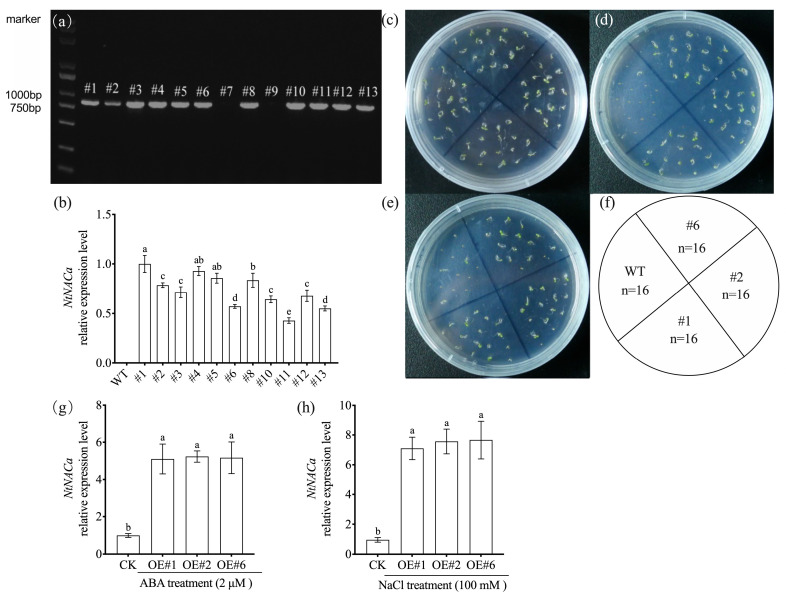
Identification and resistance testing of *NtNACa* overexpression lines. (**a**) Gel electrophoresis analysis of *Arabidopsis* lines overexpressing *NtNACa*. (**b**) Relative expression level of *NtNACa* in different lines. The germination status of *A. thaliana* on ^1^/_2_ MS medium (**c**), and ^1^/_2_ MS with 2 μM ABA (**d**) or 100 mM NaCl (**e**) within one week. (**f**) Pattern of seeds sowed. Relative expression levels of *NtNACa* in overexpression *A. thaliana* lines under 2 μM ABA (**g**) or 100 mM NaCl (**h**) treatments. #1-13 represent different *Arabidopsis* plants. CK represents *NtNACa* in overexpression *A. thaliana* lines without any treatment. Data are presented as mean values ± SD (*n* = 3). Different letters on vertical bars indicate significant difference at *p* < 0.05 using one-way ANOVA.

To elucidate the functional role of *NtNACa*, we generated transgenic *Arabidopsis* lines expressing *NtNACa* at varying levels (high—OE#1; medium—OE#2; and low—OE#6) (Figure 5a,b) and characterized their responses to abiotic stress. Germination assays on ^1^/_2_ MS medium demonstrated that while transgenic and wild-type seeds showed comparable germination under control conditions (0 mM NaCl), the *NtNACa*-overexpressing lines displayed markedly enhanced tolerance to both NaCl (100 mM) and ABA (2 μM) treatments, exhibiting significantly improved germination rates compared to wild-type controls (Figure 5c–f). Furthermore, quantitative analysis revealed that both NaCl and ABA treatments significantly upregulated *NtNACa* transcript levels in the transgenic *Arabidopsis* plants (Figure 5g,h).

**Figure 6:**
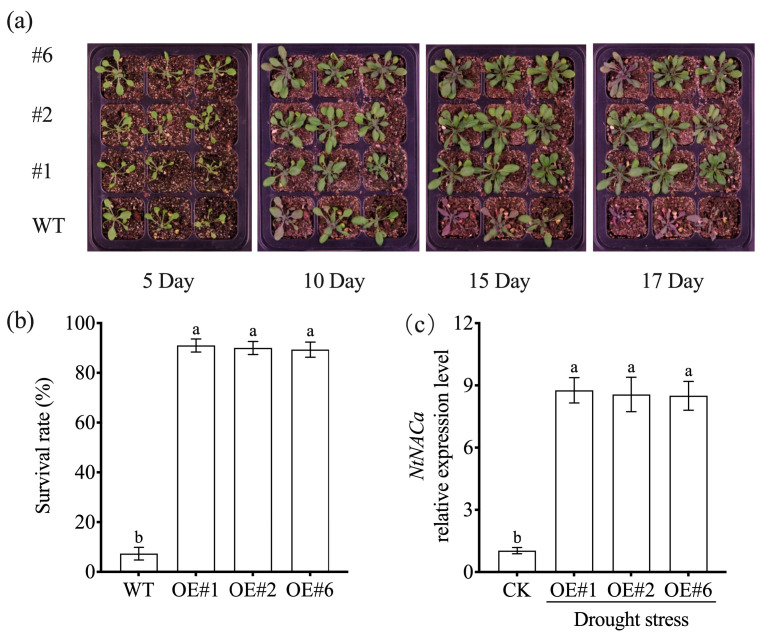
Phenotype (**a**) and survival rate (**b**) of wild-type (WT) and *NtNACa*-overexpressing *A. thaliana* lines (OE#1, OE#2, and OE#6) under drought stress. (**c**) Relative expression level of *NtNACa* in different lines under drought stress after 17 days treatment. CK represents *NtNACa* in overexpression *A. thaliana* lines without any treatment. A plant was considered ‘survived’ if it exhibited visible greening and/or produced new leaves within 7 days after rewatering; plants that remained completely wilted or turned brown were scored as dead. Data are presented as mean values ± SD of five biological replicates. Different letters on vertical bars indicate significant difference at *p* < 0.05 using one-way ANOVA.

Following 17 days of drought stress exposure, wild-type *Arabidopsis* plants displayed severe wilting phenotypes, while NtNACa-overexpressing lines (OE#1, OE#2, OE#6) maintained significantly improved leaf turgor and overall viability (Figure 6a). The transgenic lines exhibited a remarkable 89.7% survival rate compared to only 7.3% in wild-type (WT) controls (Figure 6b). Consistent with these phenotypic observations, qRT-PCR analysis confirmed strong induction of *NtNACa* expression in transgenic plants under drought stress conditions relative to unstressed controls (Figure 6c).

**Figure 7:**
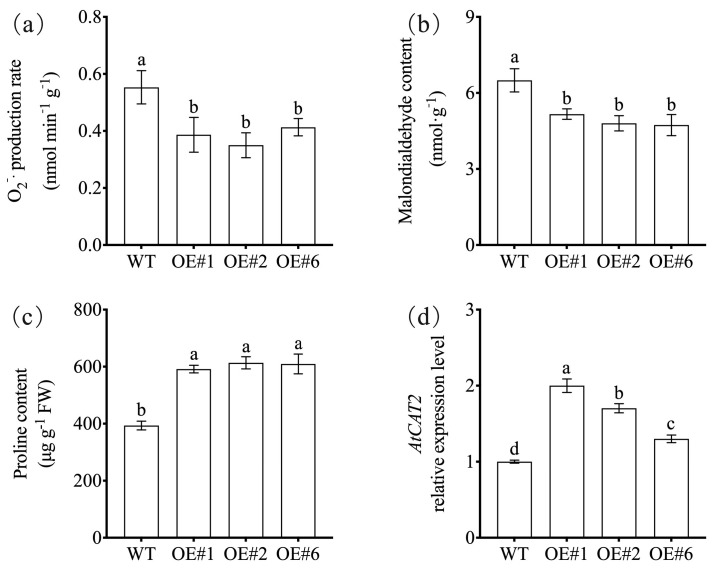
Superoxide anion (O_2_^−^·) production rate (**a**), malondialdehyde content (**b**), proline content (**c**), and relative expression of *AtCAT2* (**d**) in wild-type (WT) and *NtNACa*-overexpressing *A. thaliana* lines (OE#1, OE#2, and OE#6) under drought stress. Data are presented as mean values ± SD (*n* = 5). Different letters on vertical bars indicate significant difference at *p* < 0.05 using one-way ANOVA.

At the physiological level, the *NtNACa*-overexpressing plants displayed significantly lower O_2_^−^· production rate and MDA content compared to WT under drought conditions (Figure 7a,b). Conversely, these lines accumulated substantially higher levels of proline and exhibited elevated expression of the antioxidant gene *AtCAT2* (Figure 7c,d).

**Figure 8:**
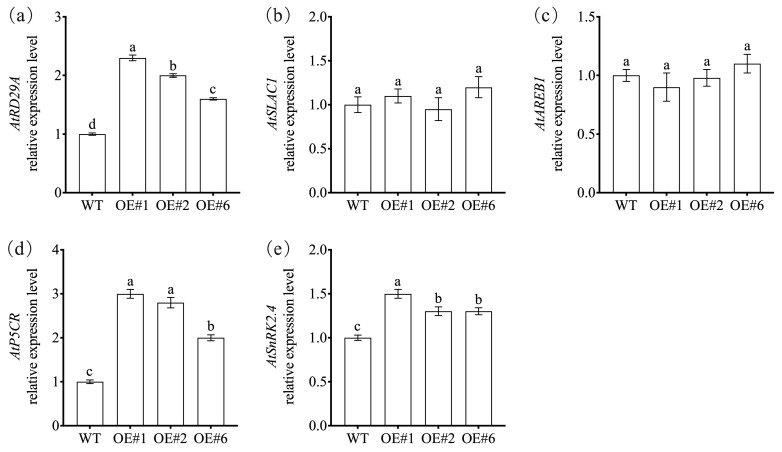
The relative expression of *AtRD29A* (**a**), *AtSLAC1* (**b**), *AtAREB1* (**c**), *AtP5CR* (**d**) and *AtSnRK2.4* (**e**) in wild-type (WT) and *NtNACa*-overexpressing *A. thaliana* lines (OE#1, OE#2, and OE#6) under drought stress. Data are presented as mean values ± SD (*n* = 5). Different letters on vertical bars indicate significant difference at *p* < 0.05 using one-way ANOVA.

Further analysis showed that key stress-responsive genes—*AtRD29A*, *AtP5CR*, and *AtSnRK2.4*—were significantly upregulated in the *NtNACa*-overexpressing lines relative to WT under drought stress (Figure 8a,d,e). In contrast, no significant differences were observed in the expression of *AtSLAC1* or *AtAREB1* between transgenic and WT plants (Figure 8b,c).

## 4. Discussion

Among various stress-responsive transcription factors, the NAC family has garnered significant research interest owing to its distinctive protein architecture and versatile regulatory roles. Extensive studies have revealed that NAC members display organ-specific expression profiles, which correlate with their functional diversification in stress adaptation [33,34]. Our investigation of ‘Yunxiang’ Narcissus identified a pronounced tissue-specific expression pattern of *NtNACa*, showing leaf-predominant accumulation, intermediate levels in roots, and minimal expression in bulbs (Figure 1b). This spatial expression bias likely reflects leaves’ role as primary environmental interfaces that necessitate rapid responses to diverse abiotic stresses including UV radiation, drought, and heat. The elevated NtNACa expression in leaves may therefore facilitate enhanced stress perception and transcriptional reprogramming. These observations align with previous reports in rice, where *OsNAC6*-driven GUS activity exhibited significantly higher intensity in leaves versus roots within 24 h [33]. Consistently, histochemical assays of transgenic *Arabidopsis* revealed stronger *ANAC072*-promoted *GUS* signals in leaves relative to roots. Salt stress treatments induced leaf-specific GUS accumulation in plants expressing *ANAC019*-, *ANAC055*-, or *ANAC072*-*GUS* fusions, whereas ABA stimulation triggered *GUS* expression in both leaves (strong) and roots (weak) of *PANAC055-GUS*/*PANAC072-GUS* lines [34]. Collectively, these findings suggest that leaf-preferential expression might represent a conserved feature of stress-responsive NAC transcription factors across plant species.

The N-terminal domain of NAC transcription factors exhibits high evolutionary conservation. Phylogenetic analysis revealed that NtNACa clusters within the monocot NAC68 clade, together with ZmNAC68, OsNAC68, SbNAC68, and SiNAC68 (Figure 1c), indicating their shared evolutionary ancestry and probable functional similarity. Sequence analysis demonstrated that NtNACa possesses all five characteristic conserved motifs (A–E) typical of NAC family proteins (Figure 2). Notably, two predicted NLSs were identified within its C-terminal transcriptional regulatory domain (Motif C and Motif D). Given that the nucleus serves as the primary site for transcription factor activity [35], we experimentally validated this prediction through subcellular localization assays, which unequivocally confirmed the nuclear localization of *NtNACa* (Figure 3). This finding is consistent with the canonical NAC protein architecture described by Puranik, et al. (2012) [36].

To investigate the dynamic expression patterns of *NtNACa* under various stress conditions and hormone treatment, we quantified its transcript levels at multiple time points post-induction. Our findings were generally consistent with established literature [37]. Previous studies have demonstrated that NAC transcription factors display remarkable functional plasticity in response to abiotic stresses. While certain members exhibit stress-specific induction patterns (e.g., the drought-responsive NAC factors in *Hevea brasiliensis* reported by Luke, et al. (2017) [38]), most NAC proteins function as broad-spectrum stress regulators capable of integrating multiple stress signals to mediate cross-tolerance mechanisms. For example, *OsNAC3* enhances ABA responsiveness and salt tolerance in rice by regulating key stress-related genes (*OsHKT1;4*, *OsHKT1;5*, *OsLEA3-1*, etc.) [39]. *OsNAC2*, targeted by *OsmiR164b*, improves drought/salt tolerance through upregulation of ABA biosynthesis genes (*OsNCED1/3*) [14]. The co-expression of the *ZmNAC111*-*ZmVPP1* module boosts drought resistance in maize by improving photosynthetic efficiency and antioxidant capacity [40]. In our study, *NtNACa* exhibited significant induction under both heat and salt stresses, with peak expression occurring at 3 h post-treatment (Figure 4a,b). This consistent temporal pattern suggests that the 3 h timepoint represents the maximal transcriptional activation phase of *NtNACa* in ‘Yunxiang’ narcissus. Notably, the ABA response exhibited a distinct biphasic pattern: transient upregulation at 3 h followed by sustained downregulation (Figure 4c). This dynamic may arise from negative feedback mechanisms (e.g., ABA-induced repressors like ABI5 attenuating NAC expression after initial activation [41]), tissue-specific signaling (as leaf-predominant *NtNACa* expression may experience different ABA kinetics than root systems [34]), or hormone concentration effects (where 100 μM ABA triggers adaptive suppression to maintain homeostasis [39]). Such complexity underscores *NtNACa*’s role as a finely tuned regulator rather than a simple on/off switch in stress responses. These results position *NtNACa* as a promising candidate for further investigation into stress response networks in Narcissus. Given the difficulty of establishing genetic transformation in Chinese narcissus, we employed a heterologous overexpression strategy by expressing *NtNACa* in *A. thaliana* to further elucidate its molecular function.

Previous research has confirmed that most heterologously expressed NAC genes do not adversely affect normal plant growth. Notable cases are *CpNAC30* from *Chimonanthus praecox* [37] and *EgNAC141* from *Eucalyptus grandis* [42], whose ectopic expression in *Arabidopsis* did not cause developmental defects. In our study, we observed comparable growth patterns between wild-type and *NtNACa*-overexpressing (OE) *Arabidopsis* plants under non-stress conditions (Figure 5c). However, under salt stress and ABA treatment, *NtNACa*-OE lines exhibited significantly higher seed germination rates (Figure 5d,e). When exposed to drought stress, transgenic plants showed markedly improved survival rates compared to wild-type controls (Figure 6a,b). Consistent with these phenotypic advantages, *NtNACa* transcript levels were substantially elevated in OE lines (Figure 5g,h and Figure 6c).

The enhanced drought tolerance conferred by *NtNACa* overexpression is underpinned by coordinated regulation of multiple canonical stress-response pathways, as evidenced by both physiological and transcriptional analyses. At the physiological level, transgenic lines exhibited significantly reduced accumulation of reactive oxygen species (ROS) and lipid peroxidation, as indicated by lower O_2_^−^· production and MDA content (Figure 7a,b), concurrent with elevated expression of the key antioxidant gene *AtCAT2* (Figure 7d). This suggests that *NtNACa* enhances cellular redox homeostasis, likely mitigating oxidative damage during water deficit. Furthermore, *NtNACa*-overexpressing plants accumulated substantially higher proline levels (Figure 7c), a critical osmoprotectant that stabilizes proteins and membranes under dehydration stress [43]. This osmotic adjustment capacity is supported at the transcriptional level by the significant upregulation of *AtP5CR*, the rate-limiting enzyme in proline biosynthesis (Figure 8d). Critically, *NtNACa* activates core components of the ABA-dependent and ABA-independent drought signaling networks. The strong induction of *AtRD29A*—a well-established marker gene responsive to both DREB-mediated (ABA-independent) and AREB/ABF-mediated (ABA-dependent) pathways [44,45]—and *AtSnRK2.4*, a SnRK2 kinase gene involved in ABA signaling [46], demonstrates that *NtNACa* interfaces with central regulatory hubs of abiotic stress response (Figure 8a,e). Notably, the expression levels of *AtSLAC1* and *AtAREB1* showed no significant changes under drought stress (Figure 8b,c), suggesting that *NtNACa* does not broadly activate the entire ABA signaling pathway but rather enhances the expression of stress-responsive genes such as *RD29A* and the accumulation of osmoprotectants. Collectively, these data position *NtNACa* not merely as a phenotypic enhancer but as a factor that enhances ROS-scavenging systems, promotes osmotic adjustment, and engages key stress-responsive gene networks—thereby providing mechanistic depth to its role in drought resilience. However, these proposed regulatory relationships remain correlative, and direct regulation by *NtNACa* requires validation by promoter-binding assays such as yeast one-hybrid (Y1H) or chromatin immunoprecipitation (ChIP).

## 5. Conclusions

This study reveals the crucial role of the *NtNACa* gene from ‘Yunxiang’ Narcissus in abiotic stress responses. The gene exhibits leaf-specific high expression, shows high homology with monocot NAC family members, possesses typical NAC transcription factor characteristics, and is localized in the nucleus. Our findings demonstrate that *NtNACa* expression is significantly induced by high temperature, salt stress, and ABA treatment. Overexpression of *NtNACa* in *Arabidopsis* enhances salt and drought tolerance, manifested by improved germination rates and increased survival rates. Mechanistically, *NtNACa* enhances drought resilience not merely through phenotypic improvement but by orchestrating a coordinated defense response: it reduces oxidative damage by reducing superoxide and MDA accumulation while upregulating the expression of antioxidant gene *AtCAT2*; it promotes osmotic adjustment via increased proline biosynthesis, correlated with elevated *AtP5CR* expression; and it activates core abiotic stress signaling hubs, including *AtRD29A* and *AtSnRK2.4*. This research provides important insights into the stress resistance mechanisms of Chinese narcissus and identifies *NtNACa* as a promising candidate gene for enhancing abiotic stress tolerance. However, its practical utility in molecular breeding requires further validation, including: (i) functional characterization in Narcissus or related geophytic monocots via stable transformation or genome editing; (ii) assessment of natural allelic diversity across ornamental cultivars; and (iii) evaluation of potential growth-flowering trade-offs under controlled and field conditions.

## Data Availability

All data is provided in the manuscript.

## Abbreviations

The following abbreviations are used in this manuscript:

TF	Transcription factor
NLS	Nuclear localization signal
ORF	Open reading frame
OE	Overexpressing
qRT-PCR	Quantitative real-time polymerase chain reaction
